# ISMB 2014—The Premier Conference for the World's Computational Biologists

**DOI:** 10.1371/journal.pcbi.1003566

**Published:** 2014-04-17

**Authors:** Christiana N. Fogg, Diane E. Kovats

**Affiliations:** 1 Freelance Science Writer, Kensington, Maryland, United States of America; 2 Executive Director, International Society for Computational Biology, La Jolla, California, United States of America

The 22nd Annual International Conference on Intelligent Systems for Molecular Biology (ISMB) will be a world-class scientific meeting that brings together computational biologists and bioinformaticians of every career stage from diverse scientific disciplines. ISMB 2014 will convene at the John B. Hynes Memorial Convention Center in Boston, Massachusetts, on July 11–15, 2014. ISMB is the flagship conference of the International Society for Computational Biology (ISCB). This unique meeting draws scientists from a broad range of fields that use computational biology and bioinformatics, including sequence analysis, comparative genomics, proteomics, structural biology, data mining, and systems biology.

ISMB 2014 is anchored by six keynote presentations from world-renowned scientists. Isaac “Zak” Kohane, the Director of the Children's Hospital Informatics Program and the Henderson Professor of Pediatrics and Health Sciences and Technology at Harvard Medical School, will be speaking on Sunday, July 13. Kohane's unique background in both pediatric endocrinology and computer science has enabled him to develop a research program that uses genomics to better understand the genetic basis of diseases, including autism and cancer. He has also developed computer systems that permit the use of information from electronic health records for genetic studies while maintaining patient privacy.

Sunday, July 13, will also feature a keynote presentation by Eugene “Gene” Myers, the 2014 recipient of the ISCB Accomplishment by a Senior Scientist Award. This award honors luminaries in the fields of computational biology and bioinformatics who have made significant contributions to these areas through research, education, and service. Myers is the Director and Tschira Chair of Systems Biology at the Max Planck Institute of Molecular Biology and Genetics in Dresden, Germany. Myers is well known for his work on developing the BLAST algorithm for sequence comparison, as well as his work on using shotgun sequencing to sequence the human genome at Celera Genomics. His research is now focused on computational bioimaging. He has developed new microscopic devices and software that are used for building 3D biological models, and these tools are providing unparalleled insights into the inner workings of cells and systems.

Michal Linial, a Professor of Biochemistry, Molecular Biology, and Bioinformatics at the Hebrew University of Jerusalem, Israel, will be a keynote speaker on Monday, July 14. Linial is the Director of the Sudarsky Center for Computational Biology and is the first female head of the Israel Institute for Advanced Studies. Her broad research activities encompass both “wet lab” projects and computational modeling, with particular interests in neuronal cell differentiation and synapse formation, proteomic analysis of membrane proteins, and functional genomics.

The 2014 winner of the Overton Prize, Dana Pe'er, is featured as a keynote speaker on Monday, July 14, as well. The Overton Prize recognizes early- or mid-career scientists working in computational biology or bioinformatics who are rising leaders in these fields. Pe'er is an Associate Professor in the Department of Biological Sciences and Systems Biology at Columbia University. Her research focuses on understanding cellular and molecular networks at a holistic level by using computational approaches to analyze complex data sets.

Robert Langer, a Professor in the Department of Chemical Engineering at the Massachusetts Institute of Technology, will give a keynote presentation on Tuesday, July 15. Langer is a prolific researcher who works on developing novel drug-delivery systems, with a particular interest in using polymers to deliver therapeutic molecules like DNA and genetically engineered proteins. Langer's innovative work was recognized most recently when he was selected as a recipient of the 2014 Breakthrough Prize in Fundamental Physics and Life Sciences.

The last keynote presentation will be given on Tuesday, July 15, by Russ Altman, a Professor of Bioengineering, Genetics, and Medicine, and Computer Science. Altman has been selected as this year's ISCB Fellows Keynote Speaker. He works on building and applying new algorithms to explore diverse topics including RNA structure, how drug efficacy is impacted by genomics, and how to model motion and dynamics of biological structures.

Beyond the keynote speakers, ISMB will be brimming with talks on cutting-edge discoveries across diverse areas. The *Special Sessions* track will run throughout the meeting and will feature hot topics that have not been featured in previous ISMB meetings. The *Highlights* and *Proceedings* tracks are also popular conference tracks that include oral presentations based on recently published papers selected through rigorous peer-review processes. The *Proceedings* papers are also published as an online-only open-access section of the *Bioinformatics* journal. The *Technology* track features presentations that showcase the use of novel software or hardware relevant to computational biologists. The *Late Breaking Research* track will also feature talks on a wide range of topics of significant interest to the bioinformatics community.

A large poster session will provide an opportunity for trainees and scientists from every career stage to present their latest research findings in a collegial and collaborative atmosphere. “Birds of a Feather” sessions and workshops will be more informal sessions that encourage discussion and collaboration. These sessions will feature such themes as bioinformatics curriculum guidelines, personalized medicine, bioinformatics core facility management, trends in digital publishing, and data analysis. The exhibit hall will showcase a wide variety of organizations and companies that are developing tools and reagents relevant to computational biologists and bioinformaticians, and attendees will be able to see some of these items in action at exhibitor presentations.

The ISCB Student Council will be organizing several high profile events throughout ISMB 2014. The annual Student Council Symposium will convene just prior to ISMB 2014 and will include talks by a keynote speaker and student presenters, as well as a poster session. Opportunities for career guidance and social events are also included. In addition, the ISCB Student Council will be coordinating an Art & Science Exhibition during the ISMB meeting that will feature images and videos of scientific material derived from research projects or artwork generated from scientific tools or methods.

Saturday, July 12, and Sunday, July 13, will be filled with substantive one- and two-day specialized meetings that precede the main ISMB meeting. Special Interest Group (SIG) and Satellite meetings will be focused on a range of topics that include but are not limited to structural bioinformatics, mass spectrometry, and regulatory genomics. Two half-day tutorial sessions will also be held on July 12 and will feature (1) Computational Metagenomics and (2) Wikipedia: WikiProject Computational Biology.

Several social events will balance out the program for ISMB 2014 and will create ample opportunities for attendees to gather together in informal settings. An opening reception is scheduled for the evening of Saturday, July 12, and poster viewing receptions are being held on both Sunday, July 13, and Monday, July 14. A World Cup viewing area will also be set up in the Exhibit Hall.

As a long-standing hub of biological and computational research breakthroughs, Boston promises to be an excellent host to ISMB 2014. Both local Boston- and Cambridge-area scientists, as well as visitors from every corner of the globe, will be showcasing diverse topics that span from personalized medicine, to machine learning in systems biology, to open-source bioinformatics software development. This must-see event has something for everyone and is an excellent destination to start your next collaboration.

